# Mechanisms and Risk Factors for Premature Ventricular Contraction Induced Cardiomyopathy

**DOI:** 10.31083/j.rcm2412353

**Published:** 2023-12-15

**Authors:** Xiaoyu Shen, Xiyao Zhu, Lingyan Zuo, Xu Liu, Mu Qin

**Affiliations:** ^1^Shanghai Jiaotong University, 200030 Shanghai, China; ^2^Department of Cardiology, Shanghai Chest Hospital, Shanghai Jiao Tong University, 200030 Shanghai, China; ^3^Shandong University of Traditional Chinese Medicine, 250355 Jinan, Shandong, China

**Keywords:** premature ventricular contraction, cardiomyopathy, mechanism, risk factor

## Abstract

Frequent premature ventricular contractions (PVCs) can cause a reversible form of cardiomyopathy in patients without structural heart disease. Because of the challenging nature of PVC-induced cardiomyopathy (PVICM), the mechanisms and risk factors for PVICM are still unclear. Based on the evidence from retrospective and observational studies, the risk factors for the development of PVICM, in addition to PVC exposure, include QRS duration, coupling interval and male sex. Based on animal models, abnormal calcium handling and cardiac remodeling may be the crucial mechanism underlying the development of cardiomyopathy. We have summarized the current knowledge on PVICM in this review. Understanding these mechanisms and risk factors is important for the diagnosis and management of this condition, which can lead to heart failure if left untreated.

## 1. Introduction

Premature ventricular contractions (PVCs) are implicated in the reversible cardiac heart failure referred to as PVC-induced cardiomyopathy (PVICM). Current guidelines and expert consensus suggest that a PVC load of 10–15% predisposes to PVICM due to impaired left ventricular function [1, 2]. There is growing clinical evidence that a PVC loading above 0.12% increases the risk of death by 31% [3]. Therefore, PVICM has become an important clinical issue that requires urgent attention. However, the exact pathophysiological mechanism of PVICM has not yet been fully clarified. Therefore, it is necessary to review the current mechanisms and risk factors associated with PVICM.

## 2. Prevalence of PVC and PVICM

PVCs, defined as early depolarization of the myocardium originating in the ventricle, are due to increased automaticity, triggered activity, or reentry. According to large cohort studies, the prevalence of PVCs ranges from 1% to 4% [4, 5], as derived from screening standard 12-lead electrocardiograms. The diagnostic capability of a 12-lead electrocardiogram (ECG) has acknowledged limits. Based on a 2-minute ECG, PVCs are present in >6% of middle-aged adults [5]. Surprisingly, the PVC prevalence is up to 69% as evaluated by 24-hour Holter monitoring in healthy adults aged 25–41 years [6]. The prevalence of PVCs is positively associated with age, in either healthy controls, or in patients with other cardiovascular diseases. The prevalence is up to 69% in individuals aged 75 years or older, while it is lower than 1% in children aged 0–11 years.

PVCs are generally considered to be benign [7], despite the reciprocal relationship between arrhythmias and cardiomyopathy. In 1988, Duffee *et al*. [8] proposed that suppression of PVCs could improve left ventricular function in patients with presumed idiopathic dilated cardiomyopathy. Since then, several studies have reported the relationship between reversible left ventricular dysfunction and frequent PVCs. The first case of radiofrequency ablation in PVICMs was reported by Chugh *et al*. [9] in 2000, who noted resolution of the dilated cardiomyopathy after eliminating PVCs by ablation. There has been increased attention to the reversible cardiomyopathy caused by PVCs.

Frequent PVCs (>5% PVCs in 24 hours) can cause left ventricular (LV) systolic dysfunction referred to as PVICM, which can be reversed by reducing or eliminating PVCs. Improvement of cardiac function was defined as LV ejection fraction (LVEF) increased by at least 10% compared to baseline (LVEF – LVEF initial ≥10%) after PVC suppression (a reduction of PVC burden ≥80% or PVC burden <1%) [10, 11]. Clinically, 54% of PVICM patients demonstrated notable improvement in LVEF, with an increase of at least 25% at 1-week follow-up compared to baseline, after effective suppression of PVCs [12]. There have been a number of studies designed to understand the epidemiology of PVICM. However, the findings were limited by the small sample size and biases of retrospective studies due to disease characteristics. Currently, based on clinical data and physician experience, most PVCs will not result in PVICM. The prevalence of PVCs with cardiac dysfunction varied from 4% to 52% [2, 13, 14, 15, 16]. In a prospective cohort study focused on the natural course of frequent idiopathic PVCs, 44% of patients had a spontaneous reduction in PVC burden to <1% per 24 hours and the median time to resolution was 15.4 months [11]. In general, PVICM is a slowly progressive disease in which LV dysfunction tends to occur after years of PVCs [15, 16].

## 3. Pathophysiological Changes in PVICM

Clinically, patients with PVICM usually have elevated brain natriuretic peptide (BNP) in contrast to patients with only PVCs. Echocardiographic measurements in PVICM patients showed LV end-systolic wall thickening, increased inner diameters during systole and diastole, suggesting systolic dysfunction and LV remodeling. Some changes, such as left ventricular dysfunction, mild cardiac fibrosis and electrical remodeling, were present in swine and canine models of PVICM, similar to those found in PVICM patients. In a PVICM canine model, eccentric hypertrophy was the typical cardiac remodeling after 12 weeks of pacing [17]. In terms of the cardiomyocyte morphology, the size of cardiomyocytes was larger in a PVICM swine model. The morphology of sarcomere, Z-line arrangement was disarrayed and the shear angles at the Z-line were reduced in the PVICM cardiomyocytes [18]. Compared to animal models, PVICM in humans usually develops over several years [16] (Table 1, Ref. [18, 19, 20, 21, 22, 23, 24, 25]).

**Table 1.** **Changes and mechanisms of PVICM based on patients and animal models**.

Structural changes					
LVEF			Decreased		
		LV mass	Increased (LV end-systolic wall thickening)		
			Larger size of CMs [18]		
		fibrosis	mild		
Hemodynamic change [19, 20]					
		stroke volume	Decreased during PVC		
		overall cardiac output	↓		
Autonomic dysregulation			extracardiac sympathetic hyperinnervation and sympathetic neural hyperactivity		
			increased coronary sinus norepinephrine levels		
Myocyte remodeling					
		T-tubules	Decreased [21]		
		Z-line	Disarrayed, shear angles of z-line reduced		
		dyad	Dyadic density decreased, JPH2 and BIN1 declined		
		Conformation of myosin heads	unknown		
Electrical changes					
	ICaL		↓ [21]		
	Ito		↓ [21]		
	IK1		↓ [21]		
			Prolonged APD and exaggerated variations [21]		
Calcium signaling alteration					
				Protein levels	Distribution
		Cav1.2	Decreased	↓ [21, 22]	LV free wall; LV
		SERCA2a	Decreased	↓ [21, 23]	LV free wall; LV
		PLN	Increased	↑ [21, 23]	LV free wall; LV
		pPLN		↓ [23]	LV
		RyR2	Increased	↑ [24], - [22]	LV
		NCX	Increased	↑ [24], - [22]	LV: shift from dyads to peripheral sarcolemma [25]
		CaMKII-α	Increased	↑ [24]	basal-lateral LV
Dayd remodeling					
		JPH2		↓ [22]	LV: dim and dispersed
		BIN1		↓ [22]	LV: dim and dispersed

↑, up-regulated; ↓, down-regulated; -, no significant change in the study. PVICM, premature ventricular contraction-induced cardiomyopathy; LVEF, left ventricular ejection fraction; LV, left ventricular; CMs, cardiomyocytes; PVC, premature ventricular contraction; JPH2, junctophilin-2; BIN1, bridging integrator-1; APD, action potential duration; Cav1.2, voltage-gated calcium channel 1.2; SERCA2a, sarcoplasmic reticulum calcium ATPase; PLN, phospholamban; pPLN, phosphorylated phospholamban; RyR2, ryanodine receptor type 2; NCX, sodium-calcium exchanger; CaMKII-α, calcium/calmodulin-dependent protein kinase II alpha; Ito, transient outward $K^{+}$ currents; IK1, inward rectifier $K^{+}$ currents; ICaL, L-type ${\mathrm{Ca}}^{2+}$ currents.

The detection of cardiac hemodynamic indicators in different pacing modes found that cardiac output was significantly reduced in ventricular demand pacing or inhibited ventricular pacing (VVI) and dual-chamber demand pacing with dual-rate responsiveness (DDDR) modes compared with the atrial demand pacing or inhibited atrial pacing (AAI) mode [19]. pulmonary capillary wedge pressure (PCWP), right atrium (RA) pressure and pulmonary artery pressure all increased, and left ventricular output index was lower in the VVI mode [19]. LVEF was significantly decreased as assessed by radionuclide angiography [19]. There are similar changes in hemodynamics in patients with PVCs. The stroke volume decreased during the premature beat [20]. Although the post-systolic enhancement effect of the next heart beat might compensate for the lost output, the overall cardiac output is lower than that of the sinus beat. With the prolonged RR interval, the left ventricular end-systolic pressure caused by post-extrasystolic potentiation (PESP) increased significantly [26].

Over 50% of PVICM patients achieved improved cardiac function and symptom remission after eliminating PVCs with antiarrhythmic drugs and ablation [12]. Treatment in PVC patients can improve LV diastolic function and left atrial function in the short term. Animal experiments found that myocardial interstitial fibrosis, autonomic dysregulation and LV mechanical dyssynchrony persist for a few weeks after eliminating PVCs, despite improvement in LV function [25]. Further studies found electrical remodeling in PVICM during sinus rhythm persists [27]. These irreversible changes might account for the higher risk of sudden death, malignant arrhythmias, and heart failure in patients who have had PVICM or PVCs [28].

## 4. Mechanism of PVICM

The underlying mechanism of PVICM remains controversial. PVICM was initially classified as a tachycardia-induced cardiomyopathy because of the reversible cardiac function associated with arrhythmias [9]. Nevertheless, heart rate did not increase substantially in patients with PVCs according to ECG and 24 h-Holter monitoring, and except for the interval PVC that can increase heart rate, most systolic PVCs are invalid heartbeats. In comparison to cardiac remodeling in animal models of atrial and ventricular contractions, it was determined that the development of PVICM was not related to tachycardia and heart rate irregularity. Previous studies have consistently suggested that probable mechanisms involved in PVICM include abnormal calcium handling, dyssynchronous ventricular contraction, autonomic dysregulation and myocardial remodeling (Table 1).

### 4.1 Electrical Remodeling and Abnormal Calcium Handling

**Fig. 1.**
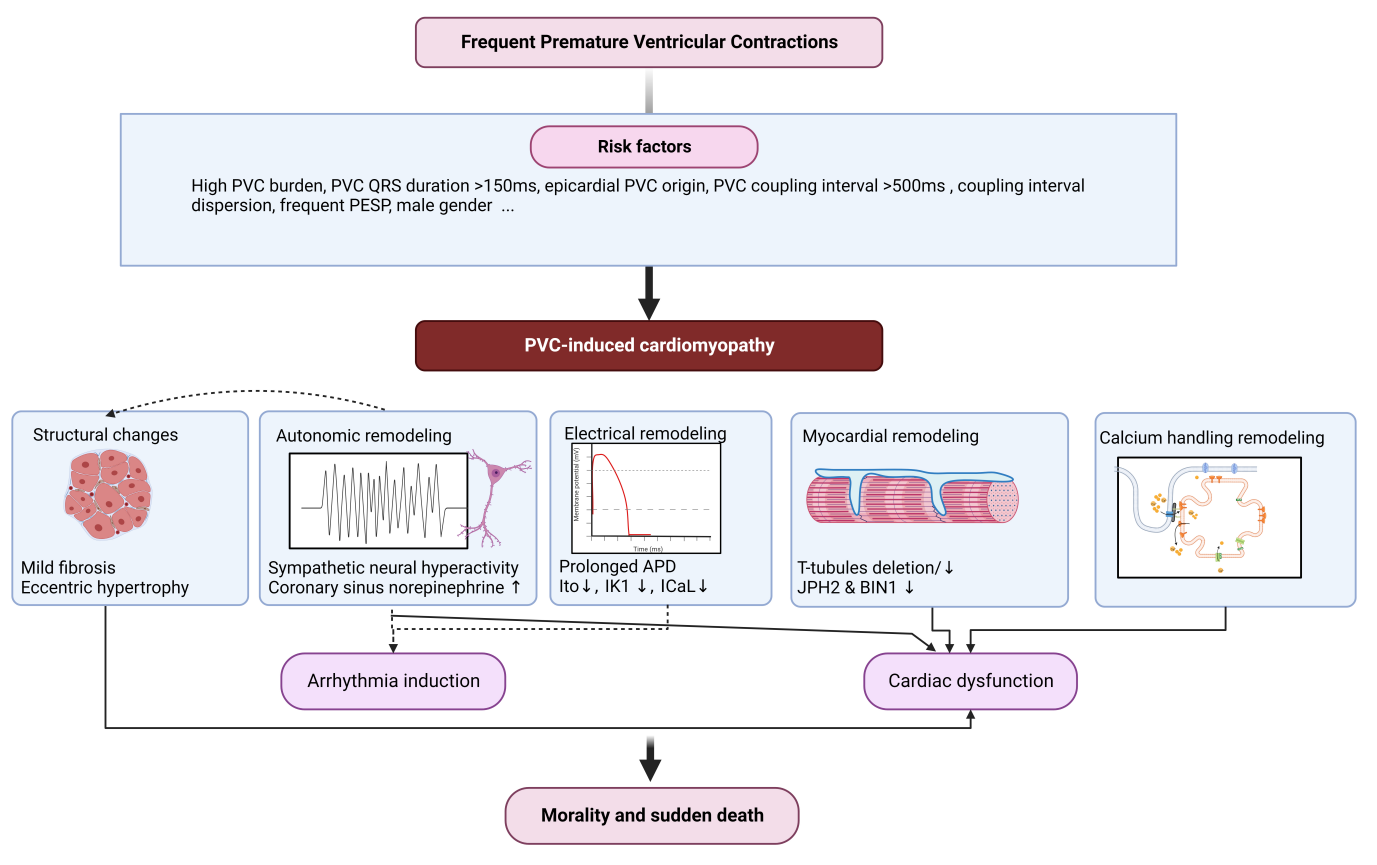
**premature ventricular contractions (PVCs) is the most common cardiac arrhythmia in patients, while some of them could develop cardiac dysfunction in the several years**. According to different characteristics in patients, risk factors for premature ventricular contraction-induced cardiomyopathy (PVICM) are complex and diverse, including PVC burden, PVC QRS duration PVC origin and sexuality. Investigation of molecular mechanisms has predominantly been studied in animal models, primarily swine and canine. Based on those models, typical tissue alterations in PVICM are mild fibrosis and eccentric hypertrophy. Besides, frequent PVCs enhance sympathetic activity, further exacerbating structural alteration in models. Substructural remodeling includes reduced T-tubules, decreased dyad intensity and Z-line arrangement disarray, leading to reduced L-type calcium currents and decreased systolic calcium transient synchrony. All of the changes might function corporately or separately, resulting in cardiac dysfunction and malignant arrhythmia, increase the risk of sudden cardiac death. PESP, post-extrasystolic potentiation; JPH2, junctophilin-2; BIN1, bridging integrator-1; APD, action potential duration; Ito, transient outward $K^{+}$ currents; IK1, inward rectifier $K^{+}$ currents; ICaL, L-type ${\mathrm{Ca}}^{2+}$ currents. Created with https://biorender.com/.

Excitation-contraction coupling is a process providing the basis for muscle contraction, in which the key effector molecule is calcium ions. Calcium-induced calcium release (CICR) is a fundamental cellular mechanism for generating and amplifying intracellular calcium signals. The excitation-contraction coupling of cardiomyocytes depends on this process. PVCs lead to prolonged action potential time of ventricular cardiomyocytes and a decrease in the density of the transient outward $K^{+}$ currents (Ito), inward rectifier $K^{+}$ currents (IK1) and L-type ${\mathrm{Ca}}^{2+}$ currents (ICaL), accompanied by decreased expression of the associated ion channel subunits [21]. Changes in ion channels may lead to abnormal repolarization of cardiomyocytes, increasing the risk of malignant arrhythmias [28] (Table 1, Fig. 1). In the swine model of PVICM, decreased sarcoplasmic reticulum (SR) calcium ATPase (SERCA2a) and increased ryanodine receptor type 2 (RyR2), sodium-calcium exchanger (NCX1), calcium/calmodulin-dependent protein kinase II alpha (CaMKII-α) and phospholamban (PLN) expression were observed [24] (Table 1). Alterations in protein expression and ion currents suggest that PVCs also lead to impaired CICR and abnormal excitation-contraction coupling, resulting in abnormal cardiac systolic function and smaller positive inotropic effects. Furthermore, voltage-gated calcium channel 1.2 (Cav1.2) protein expression and ICaL density were reduced, and Cav1.2 protein was relocated away from the t-tubules, leading to the decrease of ${\mathrm{Ca}}^{2+}$ entering the dyad gap through the opened L-type calcium channel (LTCC) resulting in the reduction in cytoplasmic ${\mathrm{Ca}}^{2+}$ concentration, reduced calcium spark events, and impairing E-C coupling in PVICM cardiomyocytes (Fig. 1). The decreased expression of SERCA2a, leading to reduced SR calcium reuptake in cardiomyocytes with PVICM, resulted not only in a reduction in ${\mathrm{Ca}}^{2+}$ concentration in the SR, but also in an increase in the cytosolic ${\mathrm{Ca}}^{2+}$ concentration, thereby inhibiting myocardial relaxation. Thus, dysfunction of the ventricular systolic and diastolic function results from decreased SERCA2a expression. Physiologically, dephosphorylated PLN interacts with SERCA and decreases SERCA calcium affinity, inhibiting calcium reuptake by the SR. In recent years it has become increasingly clear that several ${\mathrm{Ca}}^{2+}$-dependent proteins contribute to the fine tuning of E-C coupling. One of these is the ${\mathrm{Ca}}^{2+}$/calmodulin-dependent protein kinase (CaMK) of which CaMKII is the predominant cardiac isoform. PLN could be phosphorylated at Thr-17 by CaMKII, alleviating the PLN-mediated inhibition of SERCA activity and increasing the SR ${\mathrm{Ca}}^{2+}$ uptake [29]. The up-regulation of the inhibitory protein PLN aggravates the E-C coupling disorder of cardiomyocytes. The up-regulation of CaMKII-α can partially alleviate the abnormal calcium handling mediated by the up-regulation of PLN, which may be a compensatory mechanism to prevent the heart from complete systolic failure. Furthermore, CaMKII can enhance RyR2 activation, when it can both unload ${\mathrm{Ca}}^{2+}$ from the SR and induce arrhythmias in the setting of heart failure [29]. Additionally, increased NCX expression leads to increased intracellular calcium excretion, which may partially alleviate diastolic dysfunction due to reduced calcium reuptake via SR, but may lead to further reduced systolic function [30, 31, 32]. Reduced IK1 current disrupts resting membrane potentials and further enhances arrhythmias mediated by NCX upregulation [30].

Genome-Wide Association Studies (GWAS) confirm the association of genes coding for calcium handling proteins are at an increased risk of PVICM development in patients with frequent PVCs. It is hypothesized that mutations in calcium handling genes may affect calcium homeostasis, resulting in decreased sodium current and slow conduction, thereby prolonging the QRS duration [33].

### 4.2 Dyssynchronous Ventricular Contraction

Dyssynchronous ventricular contractions have long been considered to be the primary mechanism for the development of PVICM. In PVICM animal models, a linear relationship was found between the degree of LV dyssynchrony and the upregulation of CaMKII-α-mediated RyR2 phosphorylation [24], suggesting that CaMKII-α may be a particularly important mediator and a potential therapeutic target for PVICM. In a PVICM swine model, LV intra mechanical remodeling persists despite the normalization of LV systolic function 4 weeks after stopping pacing [25], which suggests that PVICM patients recovering from cardiac dysfunction should continue to be closely followed.

### 4.3 Neuromodulation

The 24-hour electrocardiogram analysis of heart rate variability in patients with idiopathic PVCs found that autonomic activities were involved in the occurrence of PVCs [34, 35], and frequent PVCs lead to increased peripheral tissue and cardiac sympathetic activity and increased coronary sinus norepinephrine levels [26]. In a swine model of PVICM, neural remodeling was characterized by extracardiac sympathetic hyperinnervation and sympathetic neural hyperactivity [36]. The neural remodeling—stellate ganglia hyperinnervation—persisted despite normalization of LV systolic function [37]. Cardiac dysfunction in PVCs may be triggered and facilitated by chronic disruption of the sympathetic-vagal balance. At an early stage, depletion of cardiac transient receptor potential vanilloid-1 (TRPV1) afferents by resiniferatoxin (RTX) improved LV systolic function and alleviated cardiac fibrosis in PVICM animals, while this improvement was not apparent at late stages, and had no effect on autonomic activity [38]. These studies suggest that TRPV1 mainly plays a role in promoting myocardial fibrosis in the early stage, and then myocardial remodeling and dysfunction are mainly affected by sympathetic imbalance, indicating that neuromodulation may play distinct roles at different stages of PVICM.

### 4.4 Myocardial Remodeling

The sliding filament theory is a classic model for explaining muscle contraction proposed by Andrew Huxley and Hugh Huxley in 1954, and is almost universally accepted [39, 40]. The cross-bridge cycling results in force production through cyclical conformational changes of myosin heads. The structure of myosin-actin interaction in the cross-bridge cycling has different characteristics and different spatial positions from thin filaments, as determined by X-ray diffractometry and scanning electron microscopy. The state of myosin heads close to the thin filament is defined as the disordered relaxed state; the super relaxed state is a low-energy metabolic state, where myosin head interacts with one another and the blocked head (BH) interacts with the lever of thick filaments, keeping the myosin head away from the filament and making it difficult to bind adenosine triphosphate (ATP) [41, 42]. It is currently believed that myosin super relaxed state (SRX) plays an important role in regulating energy utilization and cardiac contraction [43]. The cellular basis for the Frank-Starling mechanism is length-dependent activation (LDA). Calcium sensitivity increases when sarcomeres are stretched, causing increases in cardiac contractility. The mechanism of myofilament LDA derived from swine ventricular myocytes is that passive stretching converts more myosin SRX to disordered relaxed state (DRX) [44]. Changes in the balance between SRX/DRX may explain cardiac dysfunction occurring in cardiac diseases. Studies on familial hypertrophic cardiomyopathy have found that most variants are located in genes encoding myosin head and neck of filaments [45, 46, 47]. The structural changes of thick filaments change the DRX/SRX balance, and more myosin heads are in the DRX state resulting in cardiac hypercontractility and impaired diastolic function. Furthermore, Mavacamten, a myosin inhibitor that favors the closed conformation of myosin heads, achieved significant therapeutic effects in a Phase III clinical trial in obstructive hypertrophic cardiomyopathy [48]. In dilated cardiomyopathy, the aspartate-to-alanine substitution at position 94 in the regulatory light chain of myosin (RLC) encoded by the *myosin regulatory light chain 2* (*MYL2*) gene results in an increased number of SRX heads and a subsequent reduction in myocardial contractility [45]. In addition to calcium handling, experimentally, myocardial force production is also modulated by alterations of conformations of the myosin head during ischemia, hypoxia or stretching. Therefore, it is important to explore the role of DRX/SRX in reversible cardiomyopathies such as PVICM and the efficacy of myosin inhibitors in PVICM.

Studies of myocardial cytoskeletal proteins in heart failure (hypertrophic cardiomyopathy (HCM), dilated cardiomyopathy (DCM)) have suggested that cytoskeletal proteins, such as actin and desmin were significantly reduced [49, 50]. Similar changes in the actin cytoskeleton were observed in a swine model of PVICM, which was associated with Z-line arrangement disarray and played a crucial role in inefficient contractile function and cardiomyocyte remodeling [18]. This adaptive change may initially protect the heart from changes in mechanical stress to adapt to pressure changes, but long-term, there is a lack of adaptation and a decrease in the systolic and diastolic capacity of the heart. Similar alterations were observed in PVICM.

Dyads are subcellular structures of calcium signaling in cardiomyocytes, where the interconnection and connection of the plasma membrane network composed of the SR and the T tube, act as the intracellular calcium synapse of cardiomyocytes. In PVICM, the number of T-tubules is highly reduced, while the length of sarcomere and mitochondrial structure do not change significantly [22]. Decreased T-tubules diminish dihydropyridine receptor-ryanodine receptor (DHPR-RyR) coupling efficacy, which is responsible for reduced L-type calcium currents and decreased systolic calcium transient synchrony [22]. T-tubule remodeling occurs before the onset of ventricular dysfunction [51]. Previous studies suggest that the decreased expression of structural proteins such as junctophilin-2 (JPH2) and bridging integrator-1 (BIN1) is implicated in dyad formation [22, 52]. The N-terminal of JPH-2 is attached to the cell membrane through the MORN domain, and the C-terminal transmembrane structure anchors the SR, maintaining the stability of the SR and T tubular membrane structure. BIN1, as a membrane scaffold protein, plays an important role in the formation and structural maintenance of T-tubules. Decreased expression of both structural proteins will cause dyad abnormalities. It was hypothesized that increased stress on the local ventricular wall during PVCs was responsible for decreased expression of junctophilin-2 in cardiomyocytes and the remodeling of T-tubules [53] (Fig. 1).

## 5. The Risk Factors for Developing PVICM

Clinically, most frequent PVCs do not develop into a cardiomyopathy. Domestic and foreign guidelines have not clearly defined the need for eliminating PVCs to prevent cardiac dysfunction. It is still recommended that PVC patients with a high risk of PVICM undergo serial echocardiography to evaluate the changes in cardiac structure and function.

The risk factors for developing PVICM include PVC burden, QRS duration, PVC origin, interpolated PVCs, and male sex (Table 2, Ref. [15, 28, 54, 55, 56, 57, 58, 59, 60, 61, 62]). However, the effects of PVICM remain controversial.

**Table 2.** **Risk factors for premature ventricular-induced cardiomyopathy**.

	Del Carpio Munoz F *et al*. [54]	Ghannam *et al*. [55]	Kawamura *et al*. [56]	Bas e*t al*. [57]	Yokokawa *et al*. [58]	Sadron Blaye-Felice *et al*. [59]	Voskoboinik *et al*. [60]	Olgun *et al*. [61]	Billet *et al*. [62]	Limpitikul *et al*. [15]
Number of patients	17	120	51	43	113	96	39	21	17	29
PVC burden	29.3%	22%	19%	>24%	19%	26%	>20%	30%	NS	NS
PVC QRS duration	>140 ms	>150 ms	NS	>150 ms	>150 ms	NS	>160 ms	-	NS	NS
Sinus QRS duration	-	-	-	-	-	Long sinus QRS duration*	-	-	-	-
PVC origin	RV PVCs	NS	NS	NS	Epicardial PVCs	Epicardial PVCs	NS	-	NS	-
Coupling interval	-	-	Longer CI	-	-	Long CI	>500 ms	-	-	NS
CI-dispersion	-	-	115 ms (maximum-CI–minimum-CI)	-	-	-	-	-	-	+
Interpolation	-	-	-	More frequent	-	+	-	+	NS	-
PESP	-	-	-	-	-	-	-	-	High PESP	-
Male sex	NS	+	NS	+	+	+	+	NS	NS	NS

*In swine models of PVICM, sinus QRS duration increased significantly in the LV Epi PVC (*p* < 0.05) and the RVFW PVC (*p* < 0.05) groups but not in the PAC or control groups [28]. PVCs, premature ventricular contractions; PVICM, premature ventricular contraction-induced cardiomyopathy; NS, no significance; RV, right ventricular; CI, coupling interval; PESP, post-extrasystolic potentiation; LV, left ventrium; Epi PVC, epicardial origin premature ventricular contraction; PAC, premature atrial contraction; RVFW, right Ventricular Free Wall.

### 5.1 The Burden of PVC

PVC burden has been the most consistent parameter to demonstrate a relationship with the development of PVICM. Compared with PVC patients with normal ejection fraction, PVICM patients tend to have higher PVC burden (16–30% per day) [28, 54, 56, 61, 63, 64]. The lowest PVC burden resulting in cardiomyopathy was 10% [65]. Baman *et al*. [65] found that a PVC burden of >24% best separated the patient population with impaired as compared with preserved left ventricular function (sensitivity 79%, specificity 78%, area under the curve 0.89) and was independently associated with PVICM. Another study suggested that the burden threshold of >26% PVCs per day was independently associated with PVC-mediated LV dysfunction [66]. Echocardiographic results confirmed that cardiac function declines and structural remodeling of left ventricle is exacerbated with higher PVC burden. LVEF was negatively correlated with PVC burden, and cardiac diameters (left ventricular end-diastolic diameter (LVEDD) and left ventricular end-systolic diameter (LVESD)) were positively correlated [67]. Animal experiments have also indicated that canines developed PVICM when the PVC burden was over 25% [63]. The higher the PVC burden, the more severe the alterations of cardiac structure and function [63].

High PVC burden could serve as a predictor for the development of PVICM. However, it is still unclear why some patients do not develop cardiomyopathy despite a high PVC burden and why some patients appear to develop PVICM with a burden threshold of ≤10% PVCs per day.

### 5.2 PVC QRS Duration

The PVC QRS duration in PVICM patients was significantly longer than that in patients without PVICM (164 ± 20 ms vs 149 ± 17 ms; *p* < 0.0001) [58]. Yokokawa *et al*. [58] found that a PVC QRS width of 150ms had sensitivities of 80% and specificities of 52% for the diagnosis of PVICM, respectively. Patients with a longer PVC QRS duration (>150 ms) are more likely to experience a decline in cardiac function [55, 58]. Additionally, the sinus QRS duration was longer in the early stage of PVICM, and gradually prolonged with the development of the disease, although changes were minor [28]. Therefore, increased attention should be paid to the risk of heart failure in PVC patients, especially in those whose QRS duration of PVC and sinus beat is longer. Baseline QRS duration is inversely related to LVEF improvement after PVC ablation, especially in patients with a baseline QRS duration >130 ms [68].

Abnormalities of Calcium handling related proteins interrupt calcium homeostasis and are associated with prolonged QRS duration [33]. Therefore, it is thought that changes of QRS duration in PVCs suggest abnormal calcium handling in ventricular cardiomyocytes, ultimately resulting in PVICM.

### 5.3 The Origin of PVC

The site of the PVC origin has an impact on the PVC QRS width and the degree of cardiac asynchrony. LV dysfunction was observed with PVCs from all common anatomic regions of origin. In contrast, PVCs originating from the epicardium appear to cause a more pronounced LV dyssynchrony and to induce LV systolic dysfunction, with longer QRS duration [58, 69]. PVCs originating from the RV were more likely to induce cardiac enlargement and compromise cardiac function [54]. However, results in other studies were contradictory, negating the association of PVC origin with the PVICM [66, 70]. It is commonly assumed that cardiac dyssynchrony is related to the PVC origin, but some studies have found that the degree of dyssynchrony is mainly dependent on the coupling interval rather than the origin [70].

### 5.4 Coupling Interval of PVCs

In a previous study, a PVC coupling interval >500 ms was associated with abnormal LV remodeling [60]. According to a cohort study of 108 patients with frequent monogenic PVCs (of whom 22 were diagnosed with PVICM), the coupling interval in PVCIM was significantly higher than that in the control group. A PVC coupling interval of 486 ms had sensitivities of 0.789 and specificities of 0.738 for the diagnosis of PVICM [71]. Another study has shown that PVC coupling interval heterogeneity, rather than coupling interval duration, is an independent risk factor for PVICM [15]. Compared to PVC patients without cardiomyopathy, PVICM patients had significantly longer coupling interval dispersion (CI-dispersion: 115 ± 25 ms vs. 94 ± 19 ms; *p* < 0.001) [56, 72]. Longer coupling interval induced cardiac and hemodynamic dysfunction, and caused sympathetic nervous system excitation to trigger the renin-angiotensin-aldosterone system (RAAS) system, thereby causing ventricular remodeling. In addition, increase in the coupling interval duration exacerbates ventricular systolic dyssynchrony, and increasing afferent neuronal activity was observed as the PVC coupling interval increased [70]. Additionally, the variability of PVC coupling interval might exacerbate hemodynamic abnormalities and the compensatory response of the neuroendocrine system.

### 5.5 Interpolation

The burden of interpolated PVCs was higher in the PVICM patients compared with other PVC patients. Interpolation of PVCs can independently predict PVICM-induced cardiomyopathy (odds ratio 4.43, 95% confidence interval 1.06–18.48, *p* = 0.04) [61].

### 5.6 Post-Extrasystolic Potentiation

PESP is defined as a physiological phenomenon of the increase in contractility following an extrasystole, and was first proposed by Oscar Langendoff in 1885. The cellular mechanism of PESP is that calcium release from intracellular stores is increased during the post-extrasystolic heartbeat. During the premature heartbeat, the transient decrease in calcium is caused by the refractoriness of RyRs. During the post-extrasystolic beat, RyRs have recovered from inactivation, and then increased intracellular calcium stores are released from these channels, resulting in increased contractility [73]. Previous studies found a significant increase in PESP in heart failure patients, and suggested that PESP could serve as a risk predictor of cardiac dysfunction and a prognostic indicator for patients with myocardial infraction [73, 74]. Increased PESP is associated with abnormal calcium cycling induced by heart failure. Similarly, patients with PVICM had a significantly higher PESP compared to controls [62]. In animal experiments, PESP was higher than at baseline after PVICM developed in canine models [75]. Furthermore, the level of PESP at baseline had a negative correlation with LVEF, suggesting that baseline PESP at the early stage of PVCs might be a predictor for PVICM [75].

### 5.7 Male Gender

In accordance with the multi-year follow-up results of multiple retrospective and prospective studies, males are at a greater risk of PVICM than females [76]. The reasons for the sex disparity in PVICM are still unclear. Female patients with frequent PVCs are more likely to experience symptoms such as palpitation and chest tightness than males, and they more often seek medical attention [77]. Furthermore, a longer history of palpitations and asymptomatic PVCs are independent risk factors for PVICM [59].

## 6. Examination Technology of PVICM

Speckle tracking image is a relatively non-invasive cardiac function imaging technology. Compared with conventional echocardiography, it can detect early myocardial structural changes before a decline in LVEF is detected by ECG. Global longitudinal strain (GLS) is a measure of LV global function that correlates with the extent of myocardial fibrosis. GLS appears to be useful to predict changes in LV function. Additionally, GLS is considered a prognostic indicator in PVICM, based on the association between mortality and GLS levels in cardiomyopathy patients [78].

Cardiac magnetic resonance imaging (CMR) is a noninvasive examination to assess focal myocardial scar and diffuse myocardial changes. In PVC patients without structural cardiomyopathy, the presence of myocardial scar was identified by DE-CMR in 25% of patients with frequent PVCs, and it was independently associated with the development of PVICM (odds ratio 2.2; 95% confidence interval 1.3–3.7; *p* < 0.005) [79].

## 7. Treatment of PVICM 

There are several therapeutic interventions to prevent heart failure in PVC patients at high risk for PVICM. American guidelines recommend that catheter ablation is the treatment strategy for patients who experience symptoms and have decreased LV function due to frequent PVCs or who are unwilling to take antiarrhythmic drugs and for whom the antiarrhythmic drug (AAD) therapy is ineffective or the side effects of drugs are intolerable [1]. 2022 European Society of Cardiology (ESC) guidelines on ventricular arrhythmias (VA) and sudden cardiac death (SCD) recommend catheter ablation for first-line treatment for PVICM [80]. The results of clinical studies and animal experiments have confirmed that cardiac function was significantly improved when PVC burden was reduced. The degree of improvement is independent on the PVC origin. PVCs originating from the left and right hearts had similar benefits from successful rhythm control [81].

Catheter ablation therapy for PVICM patients has been reported to have an immediate post-ablation success rate of 92.5% [82] and the long-term success rate is 66%–90% [81, 83]. Catheter ablation has shown a high acute success rate, and long-term monitoring has demonstrated significant reduction in PVC burden, making it more effective than AADs [84, 85]. Furthermore, compared to AADs, radiofrequency ablation can significantly improve left ventricular ejection fraction (LVEF) in patients with PVCs (from 53% to 56%, *p* < 0.001) [84]. The success rate of catheter ablation for PVCs is dependent on the origin and morphology of PVCs. According to multicenter studies on idiopathic PVCs, PVCs originating from the right ventricular outflow tract (RVOT) have the highest success rate (93%), while epicardial PVCs have the lowest success rate (67%) [86]. PVCs located in special areas such as near the His bundle may not be amenable to ablation [87]. Complications are often related to vascular puncture [86].

There is still little effect for patients with myocardial scars identified by CMR before ablation [72]. Drug therapy should be considered for these patients who have poor postoperative outcomes or who do not receive catheter ablation, in order to reduce PVC burden if possible. Medical treatment to suppress the PVCs may include the use of beta-blockers or calcium channel blockers [80, 85] while the selection of antiarrhythmic drugs is limited by cardiomyopathy and heart failure. Attention should be paid to the long-term management of PVICM patients whose cardiac function is fully restored to normal. In congestive heart failure patients with ventricular arrhythmias, the application of amiodarone can effectively suppress ventricular arrhythmias and improve ventricular function. Unfortunately, it did not reduce the incidence of sudden death or improve survival [88]. “Dyssynchrony memory”, describing a phenomenon that LV dyssynchrony persists after PVC cessation, in the recovery period of PVICM swine, is a reminder for the need for long-term follow-up in patients with PVCs and PVIC to further our understanding of ventricular arrhythmias.

**Fig. 2.**
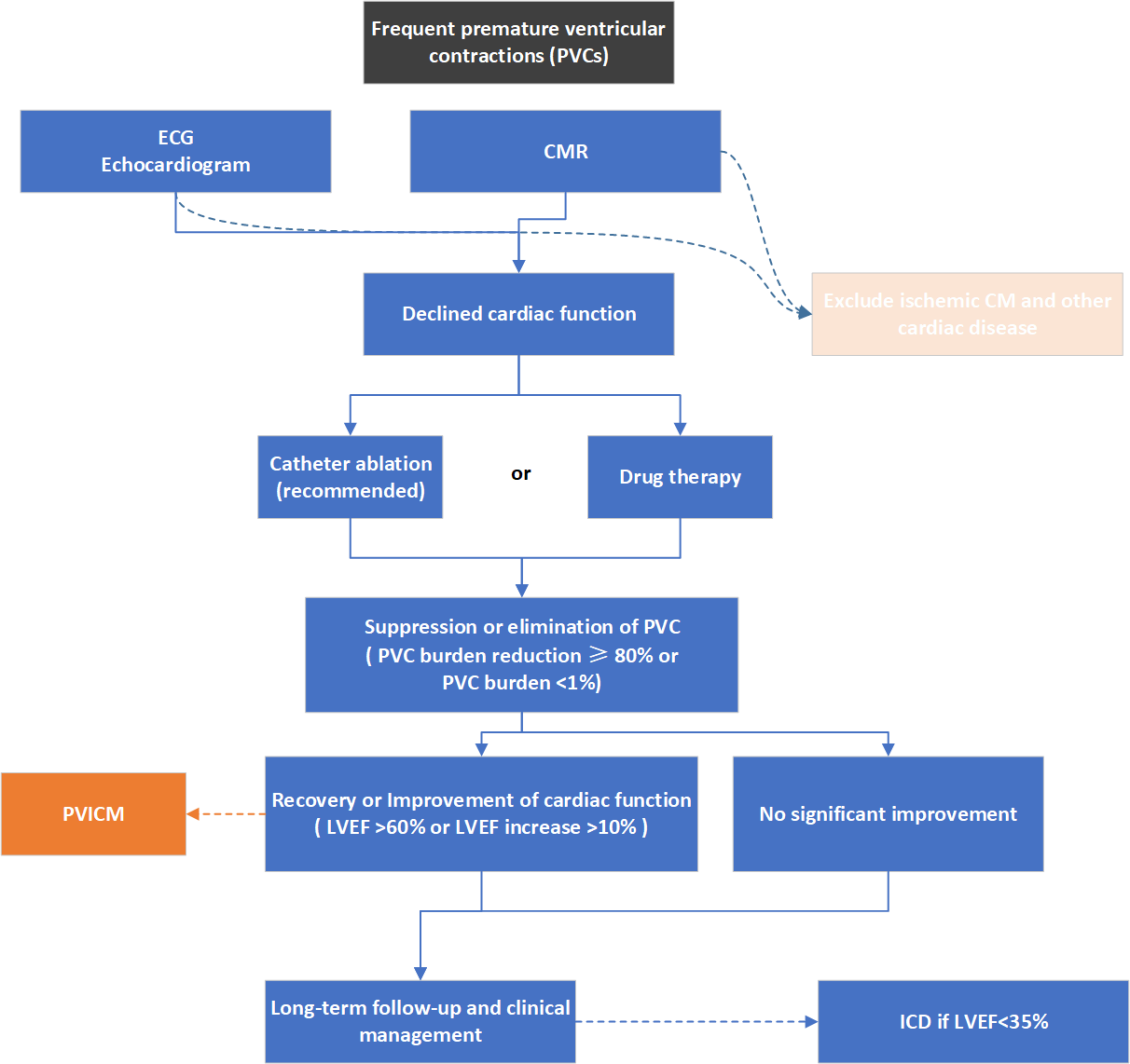
**Overview of a flow for diagnosis and treatment of PVICM**. ECG, electrocardiogram; CM, cardiomyopathy; CMR, cardiac magnetic resonance imaging; PVC, premature ventricular contraction; PVICM, premature ventricular contraction-induced cardiomyopathy; LVEF, left ventricular ejection fraction; ICD, implantable cardioverter-defibrillator.

ECG and echocardiography are essential assessment tools in the long-term management of PVICM (Fig. 2). According to the time course of recovery of LVEF in PVICM patients, the greatest improvement was observed within one week after PVC ablation [12]. Yokokawa *et al*. [89] found that most patients (around 75%) who underwent successful ablation can recover LV function within 4 months, while a few may take several years (up to 45 months) for recovery. Moreover, multicenter studies on idiopathic PVC ablation showed that around 20% of patients may require repeat ablation, primarily for PVCs originating in epicardial and papillary muscle locations [86]. In 60 PVICM patients who underwent successful ablation of PVCs, 16.7% (10 patients) experienced recurrent PVCs, resulting in PVICM recurrence [90]. Based on these findings, short-term (within one month) evaluation of treatment efficacy and recovery of cardiac function should be performed after ablation or AADs therapy, and medication should be adjusted promptly for patients who have a poor response to therapy. PVCs may indicate worsening of underlying heart disease and exacerbate heart failure. PVICM patients often experience heart failure recurrence after PVC recurrence. Therefore, follow-up with ECG and echocardiography every three to six months should be conducted to assess cardiac function and adjust anti-arrhythmic and heart failure medications in a timely manner. In addition, PVCs may trigger malignant arrhythmias such as ventricular tachycardia (VT) in patients. A clinical study showed that among 30 PVICM patients, 9 cases (36%) had ECG findings suggestive of VT, including 3 cases of sustained ventricular tachycardia [12]. Thus, prevention of sudden cardiac death (SCD) events should be emphasized in PVICM patients, and implantable cardioverter-defibrillator (ICD) implantation should be considered based on patient symptoms, ECG changes, and compliance with treatment indications [80].

## 8. Conclusions and Outlook

PVCs are common arrhythmias, and are often indicative of underlying cardiac disease. Clinical data have confirmed an emerging clinical entity of PVICM. However, many clinical studies were retrospective and non-randomized, and more prospective studies should be designed to improve the database. PVC burden is still the most robust and available risk factor for PVICM. Animal models of PVICM are still necessary to further determine the mechanism responsible for the reversible cardiomyopathy, since the association between low levels of PVC burden in humans and the development of LV dysfunction remains unclear, and awaits further investigation.
